# AQP4-IgG NMOSD, MOGAD, and double-seronegative NMOSD: is it possible to depict the antibody subtype using magnetic resonance imaging?

**DOI:** 10.1055/s-0043-1768669

**Published:** 2023-06-28

**Authors:** Diego Cardoso Fragoso, Luana Michelli Oliveira de Paula Salles, Samira Luisa Apóstolos Pereira, Dagoberto Callegaro, Douglas Kazutoshi Sato, Carolina de Medeiros Rimkus

**Affiliations:** 1 Universidade de São Paulo, Faculdade de Medicina, Departamento de Radiologia, São Paulo SP, Brazil.; 2 Universidade de São Paulo, Faculdade de Medicina, Departamento de Neurologia, São Paulo SP, Brazil.; 3 Pontifícia Universidade Católica do Rio Grande do Sul, Instituto do Cérebro do Rio Grande do Sul (InsCer), Porto Alegre RS, Brazil.

**Keywords:** Neuromyelitis Optica, Myelin-Oligodendrocyte Glycoprotein, Magnetic Resonance Imaging, Neuromielite Óptica, Glicoproteína Mielina-Oligodendrócito, Imageamento por Ressonância Magnética

## Abstract

**Background** There is clinical and radiological overlap among demyelinating diseases. However, their pathophysiological mechanisms are different and carry distinct prognoses and treatment demands.

**Objective** To investigate magnetic resonance imaging (MRI) features of patients with myelin-oligodendrocyte glycoprotein associated disease (MOGAD), antibody against aquaporin-4(AQP-4)-immunoglobulin G-positive neuromyelitis optica spectrum disorder (AQP4-IgG NMOSD), and double-seronegative patients.

**Methods** A cross-sectional retrospective study was performed to analyze the topography and morphology of central nervous system (CNS) lesions. Two neuroradiologists consensually analyzed the brain, orbit, and spinal cord images.

**Results** In total, 68 patients were enrolled in the study (25 with AQP4-IgG-positive NMOSD, 28 with MOGAD, and 15 double-seronegative patients). There were differences in clinical presentation among the groups. The MOGAD group had less brain involvement (39.2%) than the NMOSD group ( *p* = 0.002), mostly in the subcortical/juxtacortical, the midbrain, the middle cerebellar peduncle, and the cerebellum. Double-seronegative patients had more brain involvement (80%) with larger and tumefactive lesion morphology. In addition, double-seronegative patients showed the longest optic neuritis ( *p* = 0.006), which was more prevalent in the intracranial optic nerve compartment. AQP4-IgG-positive NMOSD optic neuritis had a predominant optic-chiasm location, and brain lesions mainly affected hypothalamic regions and the postrema area (MOGAD versus AQP4-IgG-positive NMOSD, *p* = 0 .013). Furthermore, this group had more spinal cord lesions (78.3%), and bright spotty lesions were a paramount finding to differentiate it from MOGAD ( *p* = 0.003).

**Conclusion** The pooled analysis of lesion topography, morphology, and signal intensity provides critical information to help clinicians form a timely differential diagnosis.

## Resumo

**Antecedentes** Há sobreposição clínica e radiológica entre as doenças desmielinizantes. No entanto, seus mecanismos fisiopatológicos são diferentes e apresentam prognósticos e demandas de tratamento distintos.

**Objetivo** Investigar as características de imagens de RM dos pacientes com doença associada à glicoproteína de oligodendrócito de mielina (MOGAD), a doenças do espectro da neuromielite óptica positivas para antiaquaporina-4 imunoglobulina G (AQP4-IgG NMOSD), e pacientes duplamente soronegativos.

**Métodos** Estudo retrospectivo e transversal para analisar as características e frequência das lesões do sistema nervoso central (SNC). Dois neurorradiologistas avaliaram consensualmente as imagens do cérebro, das órbitas e da medula espinhal.

**Resultados** Ao todo, foram incluídos 68 pacientes(25 com AQP4-IgG NMOSD, 28 com MOGAD e 15 duplo-soronegativos). Há diferenças na apresentação clínica entre os grupos. O grupo MOGAD demonstrou menor frequência de comprometimento do cérebro (39.2%) comparado com o AQP4-IgG NMOSD ( *p* = 0.002), com predomínio da distribuição das lesões nas regiões subcortical/justacortical, mesencéfalo, pedúnculos cerebelares médios e cerebelo. O grupo duplo-soronegativo demonstrou maior frequência de comprometimento do cérebro (80%), com lesões de maiores dimensões e com morfologia tumefeita, além de neurite óptica com maior extensão ( *p* = 0.006). O grupo AQP4-IgG NMOSD demonstrou neurite óptica com predomínio na região óptico-quiasmática e as lesões encefálicas acometeram predominantemente as regiões hipotalâmica e área postrema (MOGAD *versus* AQP4-IgG NMOSD *p* = 0.013). Além disso, foram observadas mais lesões na medula espinhal (78.3%) e a presença da “ *bright spotty lesion* ” foi um achado primordial para a sua diferenciação com os pacientes MOGAD ( *p* = 0.003).

**Conclusão** A análise pormenorizada das características das lesões por RM dos pacientes com doenças desmielinizantes imunomediadas fornece informações fundamentais que auxiliam os médicos no diagnóstico diferencial em um momento oportuno.

## INTRODUCTION

There have been significant advances in recent decades in understanding demyelinating diseases, notably with the discovery of autoantibodies directed against antigens located in the central nervous system (CNS). In 2004, an antibody against aquaporin-4 immunoglobulin G (AQP4-IgG) was identified, culminating in the description of a new entity called AQP4-IgG-positive neuromyelitis optica spectrum disorder (AQP4-IgG NMOSD). 1 This antigen, related to water homeostasis, has a peculiar distribution within the CNS, is located in the astrocyte endfeet, and is abundant along the ependymal surface and in the circumventricular organs 2 and the blood‒brain barrier. 3

The NMOSD diagnostic criteria comprise two different groups regardless of AQP4-IgG positivity. 4 According to Sato et al., 21.1% of AQP4-IgG-negative NMOSD patients are positive for a different antibody, myelin oligodendrocyte glycoprotein-IgG (MOG-IgG). 5 Interestingly, although this protein was initially thought to be involved in the pathophysiogenesis of multiple sclerosis (MS), its role in a new entity known as myelin oligodendrocyte glycoprotein associated-disease (MOGAD) is now recognized. 6

The MOGAD spectrum is broad and varies according to age, with acute disseminated encephalomyelitis being more common in children and recurrent optic neuritis (ON) and longitudinally extensive transverse myelitis (LETM) being more common in adults. 7 8

There is clinical and radiological overlap between MS, MOGAD, AQP4-IgG NMOSD, and antibody-negative NMOSD. 9 10 11 However, the different pathophysiological mechanisms of these diseases carry different prognoses and treatment demands, which makes their precise distinction imperative. 12 Overall, antibody-mediated demyelinating diseases, especially MOGAD, have a low prevalence, even lower than that of MS. 13 Unfortunately, the autoantibody test for these diseases is costly and not universally available. Together, these factors contribute to antibody analysis scarcity in some places, especially in developing countries.

Magnetic resonance imaging (MRI) might support the differential diagnosis between MOGAD and AQP4-IgG NMOSD, being especially important for the diagnosis of patients who fulfil the latest NMOSD criteria but are negative for AQP4-IgG and MOG-IgG (to simplify terminology, this disease will be referred to as double-seronegative). 4 6 There are some overlapping and peculiar MRI characteristics among these entities, but a consensus about typical imaging findings, mostly in the brain compartment, is still lacking. Here, we aimed to evaluate the brain, optic nerve, and spinal cord imaging features of a cohort of AQP4-IgG NMOSD, MOGAD, and double-seronegative patients to identify possible discriminators among them.

## METHODS

This is a cross-sectional retrospective study conducted between 2009 and 2018. Initially, patients aged ≥ 18 years old at the time of the study with a presumed demyelinating substrate who demonstrated some “red flags” that favored a diagnosis other than MS were selected, as described by Wingerchuk et al. 4 According to the tested serology, these patients were divided into three groups: those who tested positive for MOG-IgG were included in the MOGAD group; those who tested positive for AQP4-IgG and met the Wingerchuk criteria for NMOSD were included in the AQP4-IgG NMOSD group; and those who met the Wingerchuk criteria but tested negative for MOG and AQP4 autoantibodies, excluding an alternative diagnosis (clinical and/or laboratory), were included in the double-seronegative group. Demographic data, age at disease onset, and time between disease onset and MRI analysis were extracted from electronic medical records. The present study was approved by the local ethics committee (CAPPESQ46571015500000068), and all patients provided written informed consent.

AQP4-IgG and MOG-IgG were detected using a cell-based assay (CBA) in live HEK-293 cells, as previously reported. 5 14 The samples were collected during regular visits in our outpatient clinic and immediately centrifuged and stored at - 80°C.

According to service availability, MRI was acquired using 1.5 and 3.0 T scanners with a different head coil. The brain protocol comprised fluid-attenuated inversion recovery (FLAIR) and axial T1- and T2-weighted imaging (WI) fast spin–echo (FSE) sequences. Sagittal 3D-FLAIR sequences were obtained in 61 out of 68 exams. The other 7 exams had 3-mm-thick axial 2D-FLAIR sequences. The orbit protocol comprised axial and coronal T1WI and T2WI FSE and a coronal T2 short-tau inversion recovery (STIR) sequence. The spinal cord protocol comprised axial and sagittal T1WI and T2WI FSE and a sagittal T2 STIR sequence. Finally, T1WI FSE postcontrast imaging of the brain, spinal cord, and orbit was acquired. Magnetic resonance imaging was acquired by the time of the patients' first visit at our hospital, including 36 during an acute attack (< 30 days) and 32 in the chronic phase.

**Figure 1:**
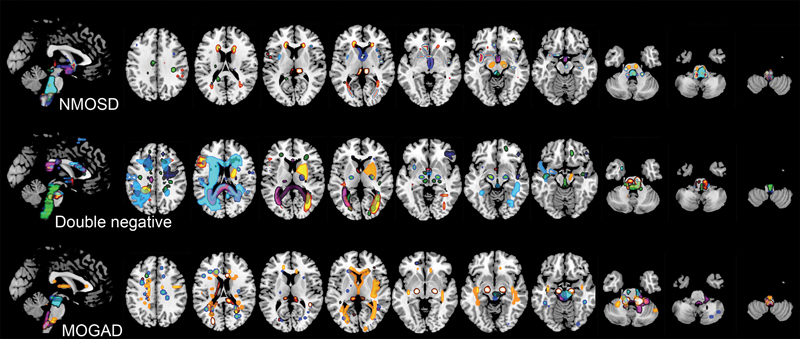
Lesion map depicts the location, morphology, and distribution of AQP4-IgG-positive-NMOSD, double seronegative, and MOGAD groups, including all patients that had brain lesions.

Two neuroradiologists with 6 (DCF) and 13 (CMR) years of experience consensually assessed all imaging data. The readers were blinded to the clinical diagnosis and serological data. Readers followed a standard assessment to rate the brain, optic nerve, and spinal cord lesions as per the Flowchart ( Supplementary Material ). Afterward, a neuroradiologist (DCF) aligned all FLAIR images into a standard template. Then, lesion masks were manually drawn for patients with brain lesions using MRIcroGL. Finally, the lesions of each group were overlaid, allowing the visualization of their distribution ( Figure 1 ).

Categorical variables are presented as absolute and relative frequencies and were compared using the Chi-squared (χ ^2^ ) test. Continuous variables are presented as the mean and standard deviation (SD) and were normally distributed and compared by analysis of variance (ANOVA). *Post hoc* tests by the Tukey method were used to identify the source of significant differences when appropriate. A *p* -value < 0.05 was considered significant. Statistical analyses were performed using IBM SPSS Statistics for MAC, version 26 (IBM Corp., Armonk, NY, USA).

## RESULTS

Twenty-five AQP4-IgG NMOSD, 28 MOGAD, and 15 double-seronegative patients were included. Most patients were in an acute or chronic phase with exacerbation (< 30 days from clinical attack), named acute disease status. There were no differences in either demographics or acute versus chronic disease status among the groups ( Table 1 ).

**Table 1:** Demographic and disease-related characteristics.

Variable		Demyelinating disease			Test
		MOGAD (28)	AQP4-IgG NMOSD (25)	Double seronegative (15)	
**Sex**	Male	11	11	5	Chi-squared 0.557
	Female	17	14	10	
**Age** (mean ± SD)		32.3 ± 2.1	38.4 ± 4.1	28.2 ± 3.0	ANOVA 0.218
**Disease status**	Active	18	14	7	Chi-squared 0.846
	Inactive	10	11	8	
Active attack to MRI* (mean ± SD)		7.8 ± 2.6	17.2 ± 2.3	19.1 ± 3.3	ANOVA 0.01
** Time elapse from last attack to MRI ^τ^** (mean ± SD)		9.5 ± 2.4	46 ± 18.3	16.3 ± 11.2	ANOVA 0.172

Abbreviations: ANOVA, analysis of variance; AQP4-IgG NMOSD, antibody against aquaporin-4-immunoglobulin G-positive neuromyelitis optica spectrum disorder; MOGAD, myelin-oligodendrocyte glycoprotein associated disease; MRI, magnetic resonance imaging; SD, standard deviation.

Notes: *days; ^τ^ months.

### Brain

The present study classified patients into those who had no evidence of brain involvement and those who had brain lesions. Eleven out of 28 (39.2%) MOGAD patients, 18 out of 25 (72%) AQP4-IgG NMOSD positive, and 12 out of 15 (80%) double-seronegative patients had supratentorial lesions. Compared with MOGAD and AQP4-IgG NMOSD patients, double-seronegative patients had more supratentorial lesions ( *p* = 0.002). Multiple comparisons showed statistically significant differences between MOGAD and AQP4-IgG NMOSD patients ( *p* = 0.004) and between MOGAD and double-seronegative patients ( *p* = 0.016).

There were no differences among MOGAD, AQP4-IgG NMOSD, and double-seronegative patients related to overall subependymal lesions when we considered patients with brain lesions. Nonetheless, the subependymal “extensive” subtype showed significant differences among the groups ( *p* = 0.010); it was present in 9.1% of MOGAD patients, 16.7% of AQP4-IgG NMOSD patients, and 58.3% of double-seronegative patients. There were significant differences between MOGAD and double-seronegative patients ( *p* = 0.017) and between AQP4-IgG NMOSD and double-seronegative patients ( *p* = 0.025).

There were no basal ganglia lesions in MOGAD or AQP4-IgG NMOSD patients. Although two double-seronegative patients (16.7%) demonstrated basal ganglia lesions, there were no differences among the groups ( *p* = 0.081).

Corpus callosum lesions were not different among the groups. However, 25% of double-seronegative patients had the “extensive” corpus callosum lesion subtype. No MOGAD or AQP4-IgG NMOSD patients had this lesion subtype ( *p* = 0.018). There were significant differences between AQP4-IgG NMOSD and double-seronegative patients ( *p* = 0.024) and between double-seronegative and MOGAD patients ( *p* = 0.048).

There were no differences in “focal” or “extensive” corticospinal tract lesions among the groups. Notwithstanding, regardless of corticospinal lesion subtype, 50% of double-seronegative patients, 18.2% of MOGAD patients, and 11.1% of AQP4-IgG NMOSD patients presented lesions ( *p* = 0.004), with significant differences between MOGAD and double-seronegative patients ( *p* = 0.005) and between AQP4-IgG NMOSD and double-seronegative patients ( *p* = 0.002).

The overall infratentorial compartment lesions did not reach any differences among the groups. Twelve of 18 AQP4-IgG NMOSD patients (67%), 1 of 11 MOGAD patients (9.1%), and 4 of 12 double-seronegative patients (33.3%) had lesions in the area postrema ( *p* = 0.016), with a significant difference between MOGAD and AQP4-IgG NMOSD patients ( *p* = 0.013).

The lesion morphology analysis showed significant differences in the “tumefactive” subtype ( *p* = 0.018). Three of 12 double-seronegative patients (25%) had “tumefactive” lesions, and no “tumefactive” lesions were observed in any patients in the other groups, with a significant difference between MOGAD and double-seronegative patients ( *p* = 0.048) and between AQP4-IgG NMOSD and double-seronegative patients ( *p* = 0.024). All other variables are included in Table 2 .

**Table 2:** Brain MRI features

Variable			Demyelinating disease Affected variable No./total No. (%)			*p* -value	Multiple comparison	*p* -value
			MOGAD (28)	AQP4-IgG NMOSD (25)	Double seronegative (15)			
**Topography**	**Supratentorial**	Supratentorial lesions	11/28 (39.2)	18/25 (72)	12/15 (80)	**0.002**	M vs. NMO+	**0.004**
							M vs. DN	**0.016**
		Juxtacortical	4/11 (36.4)	3/18 (16.7)	3/12 (25)	0.507		
		Subcortical	6/11 (54.5)	5/18 (27.8)	6/12 (50)	0.299		
		Subependymal ^†^	4/11 (36.4)	7/18 (38.8)	9/12 (75)	0.256		
		1. Extensive	1/11 (9.1)	3/18 (16.7)	7/12 (58.3)	**0.010**	M vs. DN	**0.017**
							NMO+ vs. DN	**0.025**
		2. Dawson finger	2/11 (18.2)	1/18 (5.5)	2/12 (16.7)	0.534		
		3. Nonperpendicular focal lesion	1/11 (9.1)	3/18 (16.7)	2/12 (25)	0.842		
		Third ventricle	1/11 (9.1)	3/18 (16.7)	3/12 (25)	0.617		
		Temporal lobe	2/11 (18.2)	1/18 (5.5)	3/12 (25)	0.329		
		Diencephalic	1/11 (9.1)	3/18 (16.7)	3/12 (25)	0.617		
		Thalamus	2/11 (18.2)	1/18 (5.5)	4/12 (33.3)	0.147		
		Basal ganglia	0	0	2/12 (16.7)	0.081		
		Deep gray matter ^á^	2/11 (18.2)	1/18 (5.5)	4/12 (33.3)	0.147		
		Corpus callosum ^‡^	3/11 (27.3)	2/18 (11.1)	6/12 (50)	0.082		
		1. Extensive	0	0	3/12 (25)	**0.018**	M vs. DN	**0.048**
							NMO+ vs. DN	**0.024**
		2. Oval	2/11 (18.2)	0	1/12 (8.3)	0.198		
		3. Round	1/11 (9.1)	2/18 (11.1)	1/12 (8.3)	0.968		
		4. Band	0	0	1/12 (8.3)	0.306		
		Corticospinal tract	2/11 (18.2)	2/18 (11.1)	6/12 (50)	**0.004**	NMO+ vs. DN	**0.003**
		1. Focal	1/11 (9.1)	1/18 (5.5)	4/12 (33.3)	0.093		
		2. Extensive	1/11 (9.1)	1/18 (5.5)	2/12 (16.7)	0.620		
	**Infratentorial**	Infratentorial	8/28 (28.6)	13/25 (52)	7/15 (46.6)	0.094		
		Area postrema	1/11 (9.1)	12/18 (67)	4/12 (33.3)	**0.016**	M vs. NMO+	**0.013**
		Brainstem	8/11 (72.7)	10/18 (55)	7/12 (58.3)	0.657		
		Periaqueductal	1/11 (9.1)	1/18 (5.5)	2/12 (16.7)	0.620		
		Fourth ventricle	5/11 (45.4)	3/18 (16.7)	2/12 (16.7)	0.173		
		Cerebellum	3/11 (27.3)	1/18 (5.5)	2/12 (16.7)	0.283		
		Cerebellar peduncles	3/11 (27.3)	2/18 (11.1)	1/12 (8.3)	0.393		
**Morphology**	Oval well-circumscribed		2/11 (18.2)	1/18 (5.5)	2/12 (16.7)	0.534		
	Infiltrative		5/11 (45.4)	13/18 (72)	10/12 (83.3)	0.140		
	Non-specific		4/11 (36.4)	7/18 (39)	4/12 (33.3)	0.956		
	Cavitated		2/11 (18.2)	1/18 (5.5)	4/12 (33.3)	0.147		
	Tumefactive ^§^		0	0	3/12 (25)	**0.018**	M vs. DN	**0.048**
							NMO+ vs. DN	**0.024**
	Round well-circumscribed		1/11 (9.1)	0	1/12 (8.3)	0.458		

Abbreviations: M, MOGAD; DN, double-seronegative; ANOVA, analysis of variance; AQP4-IgG NMOSD, antibody against aquaporin-4-immunoglobulin G-positive neuromyelitis optica spectrum disorder; MOGAD, myelin-oligodendrocyte glycoprotein associated disease; MRI, magnetic resonance imaging; NMO, neuromyelitis optica; SD, standard deviation; vs., versus.

Notes: Data presented in % (compared to total cases with brain lesion). The *MS-like lesion* was deﬁned as an area of focal hyperintensity on a FLAIR or T2-weighted sequence, round to ovoid, and should be at least 3 mm in their long axis. 12 ^†^ Extensive lesions (defined as > 2 cm, not perpendicular); perpendicular round focal lesions (Dawson's fingers), and nonperpendicular focal lesions; ^‡^ Extensive, Focal oval, round, or band; ^§^ Defined as lesions > 2 cm; ^á^ Thalamus + Basal Ganglia. Statistically significant differences are in bold.

Brain MRI analysis of a subgroup of patients with active or inactive disease depicted additional information ( Table 3 ). In active disease, double-seronegative patients presented more tumefactive lesions than the other groups, more deep gray matter lesions than MOGAD patients, and more corticospinal cord lesions than AQP4-IgG NMOSD patients. In inactive disease, double-seronegative patients had more infiltrative lesions than MOGAD patients.

**Table 3:** Subgroup analysis (active x inactive disease)

Disease status	Variable	*p* -value	Multiple comparison	*p* -value
**Active**	Tumefactive lesion	0.043	DN	0.076*
			DN > NMO+	0.053
	Area postrema	0.024	NMO+ > M	0.019
	Subependymal	0.046	DN > M	0.037
	Deep gray matter	0.046	DN > M	0.050
	Corticospinal tract	0.035	DN > NMO+	0.029
**Inactive**	Subependymal	0.049	NMO+ > M	0.056*
	Subependymal extensive	0.000	DN > M	0.000
			DN > NMO+	0.000
	Infiltrative lesion	0.042	DN > M	0.035

Abbreviation: DN, double-seronegative; M, MOGAD; NMO, neuromyelitis optica.

Notes: Disease status refers to less (active) or > 30 days (inactive) from clinical attack. *Tendency to statically significant differences.

Lesion distribution maps allowed the visualization of specific exciting characteristics. MOGAD and double-seronegative patients showed more subcortical and juxtacortical lesions than AQP4-IgG NMOSD patients. Double-seronegative patients showed larger lesions in the cerebral hemispheres than patients in the other two groups, which was statistically supported by the presence of more lesions with tumefactive morphology. Similarly, more lesions were observed in the basal ganglia and along the corticospinal tract in the double-seronegative group than in the other groups. AQP4-IgG NMOSD patients showed more linear lesions along the supratentorial ependymal surface, with further evidence of optic-chiasmatic and hypothalamic lesions. More lesions in the midbrain, middle cerebellar peduncles, and cerebellar hemispheres were observed in the MOGAD group than in the other groups. At the same time, medulla oblongata impairment, particularly in the area postrema, was more manifest in AQP4-IgG NMOSD patients.

### Spinal cord

Four patients did not undergo spinal MRI (1 MOGAD, 2 AQP4-IgG NMOSD, and 1 double-seronegative).

Ten out of 27 MOGAD patients (37%), 18 out of 23 AQP4-IgG NMOSD patients (78.3%), and 7 out of 14 double-seronegative patients (50%) demonstrated spinal cord lesions ( *p* = 0.012), with a significant difference between MOGAD and AQP4-IgG NMOSD patients ( *p* = 0.009).

The double-seronegative group showed more affected segments per patient than the other groups ( *p* = 0.049), with a significant difference between MOGAD and double-seronegative patients ( *p* = 0.044). However, LETM was more prevalent in AQP4-IgG NMOSD patients (94.4%) than in MOGAD (60%) and double-seronegative (85.7%) patients, with a tendency toward a difference between MOGAD and AQP4-IgG NMOSD patients ( *p* = 0.055).

Bright spotty lesions were the best discriminator between MOGAD and AQP4-IgG NMOSD patients, with a significant difference by multiple comparisons ( *p* = 0.003).

The other spinal cord variables showed no differences and are summarized in Table 4 .

**Table 4:** Spinal MRI features

Variable N		Demyelinating Disease Affected variable *n* / total *n* (%)			*p* -value	Multiple comparison	*p* -value
		MOGAD (27)	AQP4-IgG NMOSD (23)	Double seronegative (14)			
Spinal lesion		10/27 (37)	18/23 (78.3)	7/14 (50)	**0.012**	**M** **vs. NMO+**	**0.009**
Number of segments ^†^		6 [ ± 5.3]	7.6 [ ± 4.7]	12.3 [ ± 5.5]	**0.049**	**M vs. DN**	**0.044**
LETM		6/10 (60)	17/18 (94.4)	6/7 (85.7)	0.068	M vs. NMO+	0.055*
Column segment	Cervical	6/10 (60)	17/18 (94.4)	5/7 (71.4)	0.124		
	Thoracic	7/10 (70)	11/18 (61.1)	7/7 (100)	0.840		
	Lumbar	4/10 (40)	1/18 (5.5)	3/7 (42.8)	0.857		
Enhancement		5/10 (50)	7/18 (38.9)	4/7 (57.1)	0.791		
Bright spotty		0	11/18 (61.1)	3/7 (42.8)	**0.004**	**M vs. NMO+**	**0.003**
H medullar sign		4/10 (40)	1/18 (5.5)	3/7 (42.8)	0.621		
Longitudinal line		4/10 (40)	2/18 (11.1)	4/7 (57.1)	0.684		

Abbreviations: M, MOGAD; DN, double-seronegative; AQP4-IgG NMOSD, antibody against aquaporin-4-immunoglobulin G-positive neuromyelitis optica spectrum disorder; LETM, longitudinally extensive transverse myelitis; MRI, magnetic resonance imaging; NMO, neuromyelitis optica; SD, standard deviation; vs., versus.

Note: ^†^ Mean deviation within brackets; *Tendency to statically significant differences. Statistically significant differences are in bold.

### Optic nerve

Thirteen patients did not undergo orbital MRI (5 MOGAD, 5 AQP4-IgG NMOSD, and 3 double-seronegative).

The presence of ON did not reach significant differences regardless of laterality among the groups. The MOGAD group showed shorter ON and fewer affected segments ( *p* = 0.006), with a significant difference between MOGAD and AQP4-IgG NMOSD patients ( *p* = 0.010) and between MOGAD and double seronegative patients ( *p* = 0.040).

Double-seronegative and AQP4-IgG NMOSD patients had more lesions in the optic-chiasm and optic-tract segments than MOGAD patients, with a tendency toward significant differences in the optic-chiasm ( *p* = 0.053) and optic-tract ( *p* = 0.063) segments among these groups. Nonetheless, multiple comparisons analyses depicted a significant difference in the optic-chiasm segment between MOGAD and AQP4-IgG NMOSD patients ( *p* = 0.048). The other optic nerve variables showed no differences and are summarized in Table 5 .

**Table 5:** Orbital MRI features

Variable N		Demyelinating disease Affected variable *n* / total *n* (%)			*p* -value	Multiple comparison	*p* -value
		MOGAD (23)	AQP4-IgG NMOSD (20)	Double seronegative (12)			
Neuritis		16/23 (69.5)	14/20 (70)	10/12 (83.3)	0.660		
	Unilateral	10/16 (62.5)	7/14 (50)	6/10 (60)	0.087		
	Bilateral	6/16 (37.5)	7/14 (50)	4/10 (40)			
Number of segments		1.7 [ ± 1.4]	3.7 [ ± 2.8]	3.6 [ ± 2.3]	**0.006**	**M** **vs. NMO+**	**0.010**
						**M vs. DN**	**0.040**
Segment	Intraorbital	14/16 (87.5)	12/14 (85.7)	9/10 (90)	0.134		
	Canalicular/prechiasmic	13/16 (81.2)	13/14 (92.8)	10/10 (100)	0.093		
	Optic-chiasm	2/16 (12.5)	8/14 (57.1)	6/10 (60)	0.053*	**M vs. NMO+**	**0.048**
	Optic-tract	1/16 (6.2)	7/14 (50)	3/10 (30)	0.063*	M vs. NMO+	0.052*
Extension	< 50%	10/16 (62.5)	5/14 (35.7)	4/10 (40)	0.472		
	> 50%	6/16 (37.5)	9/14 (64.3)	7610 (60)	0.720		
Perineuritis		4/16 (25)	1/17 (5.9)	2/10 (20)	0.789		

Abbreviations: M, MOGAD; DN, double-seronegative; AQP4-IgG NMOSD, antibody against aquaporin-4-immunoglobulin G-positive neuromyelitis optica spectrum disorder; MRI, magnetic resonance imaging; NMO, neuromyelitis optica; vs., versus.

Notes: *Tendency to statically significant differences. Statistically significant differences are in bold.

## DISCUSSION

Current knowledge of autoantibody-mediated demyelinating diseases has allowed the demonstration of the presumed initial pathophysiologic mechanism of each known nosological entity. Antibodies against AQP4, predominantly located in astrocyte endfeet, disrupt local homeostasis, leading to astrocyte destruction and ultimately promoting secondary demyelination, which are critical findings of NMOSD. 12 15 In contrast, MOGAD is a primarily inflammatory demyelinating disease, as their antibodies target a protein expressed on the outer myelin surface in the CNS. 15 Although pathophysiological mechanisms are different, patients from both groups can have overlapping imaging findings. The possibility of an undiscovered secondary factor common to both entities could contribute to understanding this intersection. 16

We investigated the MRI hallmarks of MOGAD, AQP4-IgG NMOSD, and double-seronegative patients. In line with previous reports, 13 17 MOGAD patients had a higher predilection for ON (68.2%), followed by spinal cord (38.5%) and brain (37%) lesions, while the leading clinical phenotype of AQP4-IgG NMOSD patients was spinal cord (75%), followed by brain (73.1%) and ON (71.4%). Regarding the double-seronegative clinical phenotypes, we noted a different presentation: ON was the leading phenotype (78.6%), followed by brain (76.5%) and spinal cord (50%) lesions.

Studies have shown that patients with autoantibody-mediated demyelinating diseases have more brainstem lesions than MS patients. 16 18 19 We did not include MS patients in our cohort, but brainstem lesions were a common and universal finding in the analyzed CNS demyelinating diseases.

Topographical lesion analysis demonstrates essential differences. Lesions in the area postrema are a characteristic finding in the differentiation between AQP4-IgG NMOSD and MOGAD patients, which is consistent with other articles, 18 20 21 supporting their inclusion in the Cacciaguera criteria for the differentiation of these diseases. 19

Compared with AQP4-IgG NMOSD patients, MOGAD patients had fewer brainstem lesions, 18 similar to our results. Analysis of the lesion distribution showed that MOGAD patients had more lesions in the midbrain, middle cerebellar peduncles, and cerebellar hemispheres than their counterparts. Our study includes a small MOGAD sample, which might be a limitation when generalizing this finding. Notwithstanding, MOGAD is a rare disease, being less prevalent than MS and AQP4-IgG NMOSD, and small samples are also a limitation to several other previous MOGAD studies. Nevertheless, the characteristics of middle cerebellar peduncle lesions deserve special attention, as they have been considered a discriminating factor between MOGAD and AQP4-IgG NMOSD. 21 In the future, multicenter studies are required to confirm this hypothesis.

The MOGAD patients had fewer supratentorial lesions than AQP4-IgG NMOSD and double-seronegative patients, regardless of disease activity. In our cohort, lesions in MOGAD patients were predominantly peripherally (subcortical and juxtacortical) located, whereas those in AQP4-IgG NMOSD patients were more centrally located, mostly in the hypothalamic-chiasmatic region. Several studies found significant differences in this affected topographical region between MOGAD and AQP4-IgG NMOSD patients, with the former showing more lesions in the subcortical/juxtacortical location. 19 20 21 Double-seronegative patients demonstrated a distinct lesion distribution pattern, with lesions situated both peripherally and centrally.

Subependymal lesions might offer additional substrate information to differentiate these diseases. A recent study demonstrated that AQP4-IgG NMOSD patients presented more lesions adjacent to the lateral ventricles than MOGAD patients. 16 Furthermore, lesions in the corpus callosum occurred in all groups. However, double-seronegative patients showed more “extensive” subependymal lesions than MOGAD patients and more “extensive” corpus callosum lesions than MOGAD and AQP4-IgG NMOSD patients. These findings are in line with the morphological lesion analysis and the interpretation of the lesion distribution map, as double-seronegative patients had more aggressive characteristics from all groups, with more extensive lesions and a tumefactive pattern.

An explanation for the presence of a lower number of brain lesions in MOGAD patients could be the temporal evolution of their lesions. A recent study showed that brain lesions in MOGAD patients tend to resolve more completely than those in AQP4-IgG NMOSD patients. 22 This finding should be accounted for in our analysis, as some patients underwent MRI in the chronic phase of the disease. This information has additional clinical relevance. In a hypothetical patient with a brain lesion suggestive of a non-MS-type demyelinating lesion, a sequential MR exam could help to differentiate antibody-related diseases.

Lesions in the basal ganglia and along the corticospinal tract could also reflect the more severe neurological impairment observed in double-seronegative patients. These findings are divergent in the literature and should be carefully analyzed due to the number of patients included. 16 20 Corticospinal tract involvement has been described as a hallmark of NMOSD patients. 2 However, we demonstrated that corticospinal lesions also occurred in MOGAD patients more frequently than in AQP4-IgG NMOSD patients. In a study comparing MOGAD and AQP4-IgG NMOSD patients, Chen et al. showed that MOGAD patients had approximately 2.5 times more lesions in the internal capsule than AQP4-IgG NMOSD patients. 21

Compared with MOGAD patients, AQP4-IgG NMOSD patients had more spinal cord lesions, with the cervical segment being most frequently involved, similar to other studies. 7 12 Although conus medullaris involvement has been reported as a hallmark of MOGAD patients, 23 our cohort of Brazilian patients did not show differences in this topography among the groups, similar to a Chinese study. 21

Double-seronegative patients exhibited LETM with a higher number of affected segments than MOGAD patients. This finding must be analyzed with caution, given that the number of affected segments changes throughout the course of MOGAD and NMOSD, 24 with MOGAD lesions tending to resolve in the chronic phase. 22

Bright spotty lesions were a characteristic finding of NMOSD patients, especially AQP4-IgG patients. In cases with nonspecific brain lesions or where the analysis of autoantibodies is difficult or unavailable, the detection of a bright spotty lesion might have paramount relevance.

The optic nerve is the primary target of autoantibody-mediated demyelinating diseases. 10 18 20 Similar to cerebral and spinal cord involvement, double-seronegative patients have also demonstrated more extensive lesions of the optic pathways, with more segments affected and predominantly in the intracranial segments. Compared with MOGAD patients, AQP4-IgG NMOSD patients also had more frequent lesions in the optic tracts and chiasm, findings already supported in the literature. 12 In our cohort, perineural enhancement did not allow differentiation among the diseases, contrary to what was previously reported. 24

**Figure 2:**
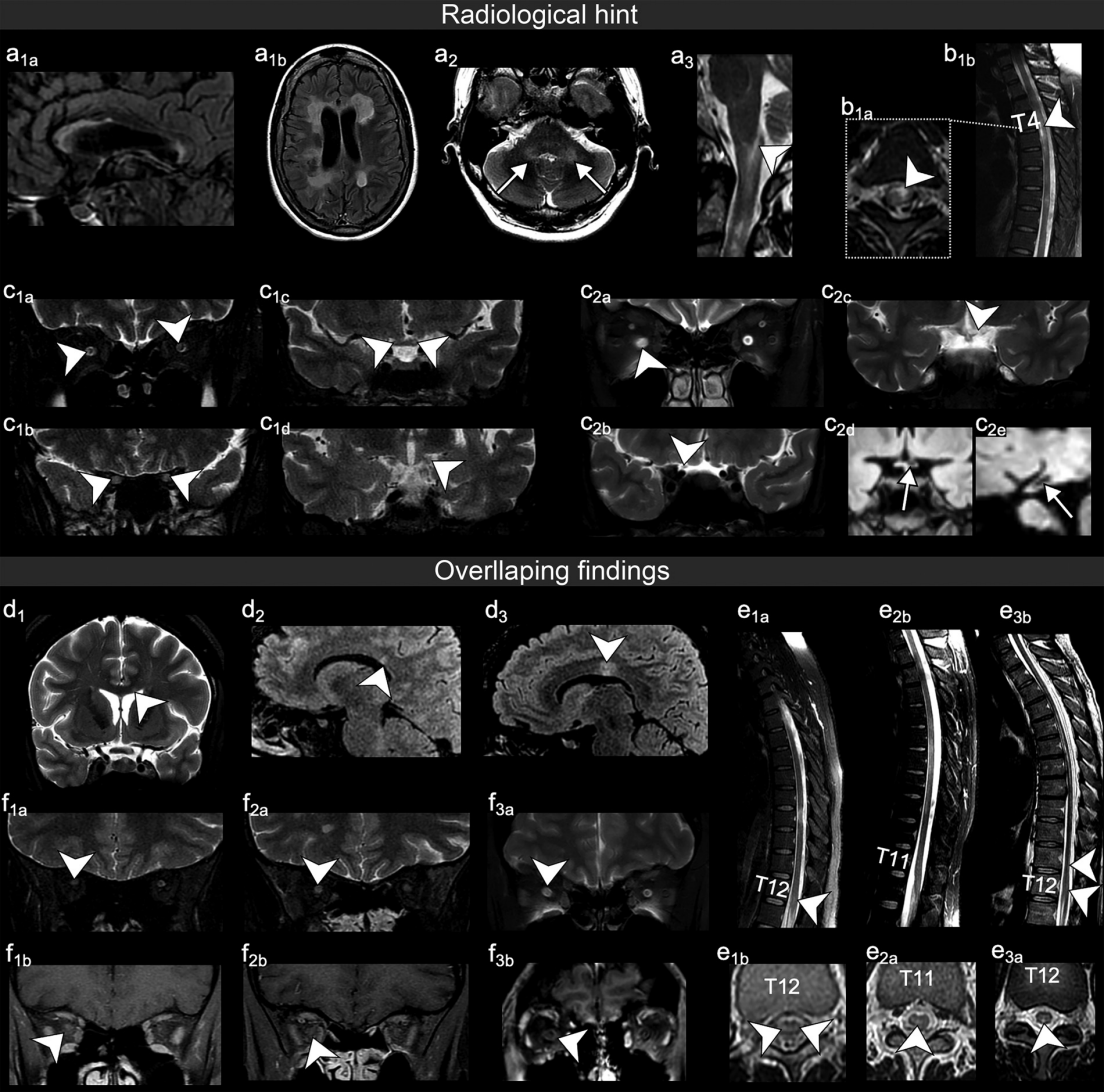
Radiological guide ;- characteristics of CNS lesions that are typical ( (hint)) and those that might be depicted (‘overlap’) in any of the antibody-mediated disease. (a1a) Extensive corpus callosum and (a1b) tumefactive lesions might be indicative of Double-seronegative. (a2) Middle cerebellar peduncle lesions, suggestive of MOGAD. (a3) Area postrema and (b1) bright spotty lesion, suggestive of AQP4-IgG-positiveNMOSD. (c1 and c2) Long ON, notably in the intracranial segments, might suggest Double-seronegative and AQP4-IgG-positive-NMOSD. Arrows in c2d and c2e demonstrated a cavitated left chiasm and tract. Corpus callosum lesion could be found in Double-seronegative (d1), MOGAD (d2), and AQP4-IgG-positive-NMOSD (d3). Conus medullary lesion could be found in Double-seronegative (e1), MOGAD (e2), and AQP4-IgGpositive-NMOSD (e3). Perineuritis could be found in Double-seronegative (f1), MOGAD (f2), and AQP4-IgGpositive-NMOSD (f3).

Figure 2 illustrates the CNS lesion characteristics of our cohort that are typical and those that might be depicted ("overlap") in any of the antibody-mediated studied diseases.

Considering all these aspects, we wondered whether MRI topographical and morphological differences among MOGAD, AQP4-IgG NMOSD, and double-seronegative NMOSD may be partially explained by differential antigen expression in specific areas. 2 12 18 Indeed, AQP4 lesions are frequently encountered in areas with high AQP4 expression (ON, periependymal, periaqueductal, hypothalamic, and spinal cord). 20 Data on MOG expression within the CNS are still lacking, but the peripheral location near the cortex has been observed more frequently than their counterparts. 19 Double-seronegative patients are an undefined entity. One could argue the possibility that double seronegativity could represent a MOGAD or AQP4-IgG NMOSD disease with undetectable or negative antibodies due to limited test sensitivities. However, the different clinical presentation compared with the other groups may be partially explained by a different inflammatory cascade or undiscovered autoantibodies. 5 A recent international randomized, double-blinded, placebo-controlled study (phase II and III) demonstrated different therapeutic responses among MOGAD, AQP4-IgG NMOSD, and double-seronegative patients using specific immunopathogenic targets, supporting a distinct mechanism among them. 25 Double-seronegative patients showed more extensive brain, ON, and spinal cord lesions.

Prospective and histopathological studies will be needed to investigate whether this disease group's distinct substrate promotes more extensive damage to the CNS, with an intersection pattern between MOGAD and AQP4-IgG NMOSD.

Our study has some limitations. First, it is a cross-sectional retrospective study with a small sample, which may not reflect the precise lesion distribution and morphology. These diseases are not prevalent, with the same limitation occurring in most studies. Thus, prospective and multicenter studies are encouraged to reduce potential selection biases and to respond with greater statistical power to these questions. Second, patients were at different stages of the disease. Some MOGAD patients might have demonstrated resolution of CNS lesions more often than AQP4-IgG NMOSD patients. Therefore, follow-up MRI exams, including moments of acuteness and intercrisis, may provide an additional underpinning to reinforce our findings. This is one of the most critical hints that could help neurologists form the correct diagnosis in clinical practice.

Our study provides further evidence that MOGAD, AQP4-IgG NMOSD, and double-seronegative patients have distinct MRI features. The pooled analysis of lesion topography, morphology, and signal intensity provides essential information to help clinicians make an early differential diagnosis.
