# Supplementary material for: AQP4-IgG NMOSD, MOGAD, and double-seronegative NMOSD: is it possible to depict the antibody subtype using magnetic resonance imaging?
Source: Arq Neuropsiquiatr. 2023 Jun 28;81(6):533–43. doi: 10.1055/s-0043-1768669 (PMC10306980; doi:10.1055/s-0043-1768669)
Supplement: Supplementary file 1 — Supplementary Material [file 10-1055-s-0043-1768669-s220252.pdf]

## SUPPLEMENTARY MATERIAL

### Flowchart - MRI assessment

**I. Brain**

*I.I - Topography*

1. Supratentorial
   (a) Juxtacortical (b) Subcortical (c) Subependymal† (d) Third ventricle (e) Temporal lobe (f) Corpus callosum‡
2. Infratentorial
   (a) Postrema area (b) Brainstem (c) Periaqueductal (d) Fourth ventricle (e) Cerebellum
3. Corticospinal tract
   (a) Focal (b) Extensive

*I.II – Morphology*

(a) Oval well-circumscribed (b) Infiltrative (c) Non-specific (d) Cavitated (e) Tumefactive§ (f) Round well-circumscribed

**II. Optic nerve**

*II.I – Laterality*

(a) Unilateral (b) bilateral

*II.II – Segments*

(1) Intraorbital (2) Canalicular / Pre-chiasm (3) Optic-chiasm (4) Optic-tract

II.III – Orbital perineural enhancement

**III. Spinal cord**

*III.I – Location*

(a) Cervical (b) Thoracic (c) Conus medullaris

*III.II – Number of involved segments*

*III.III – LETM**

*III.IV – Bright spotty*$^{\alpha}$

*III.V – Spinal line or "H" spinal cord sign*$^{\tau}$

### Notes:

I – Brain

The *MS-like lesion* was defined as an area of focal hyperintensity on a FLAIR or T2-weighted sequence, round to ovoid, and should be at least 3 mm in their long axis[1].

† - extensive lesions (defined as > 2 cm, not perpendicular); perpendicular round focal lesions (Dawson's fingers), and no-perpendicular focal lesions.

‡ - Extensive, focal oval, round, or band.

§ - Defined as lesions > 2 cm.

II – Optic nerve

The approximate lengths of the ON lesions were then scored between 1 and 4 based on the numbers of ON-involved subsegments (segment 1: intraorbital; segment 2: canalicular/pre-chiasm; segment 3: optic-chiasm; segment 4: optic-tract).

III Spinal cord

$\alpha$ - The *bright spotty lesions* are designated as spotty lesions with marked hyperintensity on T2-weighted sequences, even with higher signal intensity than the surrounding cerebrospinal fluid.

* - *Longitudinally extensive transverse myelitis is a contiguous spinal cord lesion extending for three or more segments.*

$\tau$ - *T2 hypersignal confined to gray matter in a sagittal line and forming an axial "H-sign"*[2].

## References

1. Filippi M, Preziosa P, Banwell BL, et al (2019) Assessment of lesions on magnetic resonance imaging in multiple sclerosis: practical guidelines. Brain 142:1858–1875. https://doi.org/10.1093/brain/awz144
2. Dubey D, Pittock SJ, Krecke KN, et al (2019) Clinical, Radiologic, and Prognostic Features of Myelitis Associated With Myelin Oligodendrocyte Glycoprotein Autoantibody. Jama Neurol 76:301–309. https://doi.org/10.1001/jamaneurol.2018.4053

Arquivos de Neuro-Psiquiatria © 2023. Academia Brasileira de Neurologia. All rights reserved.
